# Experts’ content validation of the parosmia, phantosmia, and anosmia test (PARPHAIT): A qualitative study

**DOI:** 10.1371/journal.pone.0329108

**Published:** 2025-08-05

**Authors:** Annelin Espetvedt, Siri Wiig, Kai Victor Myrnes-Hansen, Daniel Adrian Lungu

**Affiliations:** 1 Department of Quality and Health Technology, Faculty of Health Sciences, University of Stavanger, Stavanger, Norway; 2 SHARE – Centre for Resilience in Healthcare, Faculty of Health Sciences, University of Stavanger, Stavanger, Norway; 3 The Norwegian School of Hotel Management, Faculty of Social Sciences, University of Stavanger, Stavanger, Norway; Tehran University of Medical Sciences, IRAN, ISLAMIC REPUBLIC OF

## Abstract

**Background:**

The parosmia, phantosmia, and anosmia test (PARPHAIT) has previously been developed as a tool for capturing quantitative and qualitative symptoms of olfactory dysfunction. Its content validity was evaluated in a patient sample, from a statistical point of view through an exploratory factor analysis, and now in a panel of experts. Based on these evaluations, we present the most recent version. The aim of this study was to evaluate the content of the novel PARPHAIT in an expert panel.

**Methods:**

This was a qualitative interview study with experts in the field of olfaction. The study was done in an international research community on olfactory dysfunction. Thirteen participants (mean age 49.7, 53.8% men) with expertise in the field of smell were interviewed about PARPHAIT’s content, format, and applicability. Participants were selected based on their experience in the field of smell and invited to a digital interview.

**Results:**

Suggested improvements of PARPHAIT were provided and evaluated. Alterations were done to the formulation of items and introductory text (i.e., instructions and definitions), as well as aspects covered, and the structure and design of the questionnaire.

**Conclusions:**

PARPHAIT was considered a clear, user-friendly tool suitable for a clinical assessment context. Improvements were made based on experts’ feedback, leading to a final version of the tool. However, some aspects of PARPHAIT remain open for consideration (e.g., response and scoring design) and more work remains to reach consensus on how the PARPHAIT best can capture symptoms of olfactory dysfunction.

## Introduction

The COVID-19 pandemic brought a range of symptoms, including the loss and change of smell. This led to increased attention to olfactory (dys)function, which is not restricted to the pandemic, but includes other aetiologies like head trauma, Parkinson’s disease, and other types of viral infections [1]. In a previous review [2], we investigated the tools used to assess such symptoms in a COVID-19 population. Our findings highlighted the heterogeneity of tools and study methodology, and emphasised the unmet need of a standardised, validated tool that captures an overall symptomatic picture whilst also accounting for specific phenomena (i.e., the loss of smell, parosmia, and phantosmia). Parosmia occurs when an odour smells differently than normal (with regards to quality and/or valence) and phantosmia manifests as an odour appearing in the absence of an odour source, respectively. Especially phantosmia seems difficult to capture, as little focus has been given this in the tools available.

Most of the tools identified in the aforementioned review relied on patients’ self-report, and the greater part focused primarily on general or quantitative symptoms of smell (e.g., [3,4]). Hence, qualitative symptoms were assessed using only a few questions. Tools that paid more attention to parosmia and phantosmia had limited or missing validity and reliability analyses (e.g., [5–7]). Some tools assessed the mere presence of qualitative symptoms [8–11], while others also accounted for potential triggers and temporal aspects [5,12]. Moreover, we identified differences in the applicability (e.g., administration of measurement tools), clarity (e.g., the wording of questions and descriptions), format (e.g., the length and number of items included, the response design, the calculation of scores), and aspects not covered (e.g., asking only about the presence of symptoms and not aspects like duration and triggering factors).

These findings and identified gaps in current tools motivated the development of the Parosmia, phantosmia, and anosmia test (PARPHAIT) [13]. We included items addressing the presence of phantosmia and parosmia, duration, frequency, intensity, and potential triggers. During the development of PARPHAIT, we invited 165 participants who experienced qualitative olfactory symptoms to complete and evaluate PARPHAIT. After the initial evaluation of its content and format we conducted an exploratory factor analysis (EFA) and obtained a shorter version of PARPHAIT (S1 File). To assess the quality and content of the shorter version, the content validation was assessed by experts in the field. Experts’ feedback can provide support or opposition to factors and items suggested following the EFA, and further refinements can be done. Experts’ perspectives are crucial, as clinicians and researchers will be applying the scale and use the information to diagnose olfactory dysfunction.

### Research question, aim and objective

In this study, we ask the following research question: How suitable is the content and format of PARPHAIT in assessing olfactory dysfunction?

To answer this question, we aimed to evaluate the content validity of PARPHAIT in a sample holding expertise in olfactory research and practice. The objective was to assess length, relevance, clarity, and applicability of the tool, and based on the data collected and analysed, PARPHAIT was edited and improved accordingly.

## Methods

PARPHAIT’s content validity was assessed in a group of experts in a two-phase digitally based qualitative study. PARPHAIT has previously been addressed quantitatively [13], to which a qualitative methodology will complement our previous findings. The first phase involved experts’ review of PARPHAIT, followed by the second phase of receiving their feedback during individual, digital interviews (using Microsoft Teams, version 1.6.00.31801, and Zoom, version 5.17.11).

### Recruitment and sample

Sampling was a continuous process happening in parallel with data collection and -analysis until data saturation was reached. Sampling started on the 14^th^ of November 2023 and was completed on the 8^th^ of February 2024. The population of interest were international experts on olfactory dysfunction, most of which had experience with both quantitative and qualitative symptoms. Because the expertise within the field of smell is limited, we decided to include experts irrespective of geographical residence. Expertise included – but was not restricted to - ear, nose, and throat (ENT) clinicians, researchers, and psychologists. The process of choosing the expert sample involved a comprehensive search through research networks, authors of relevant papers, and word of mouth, followed by contacting each of them by e-mail.

The study was approved by Sikt, the Norwegian Agency for Shared Services in Education and Research (reference ID 282584, approved 31.10.2023). Invited experts received an e-mail containing the interview guide (S2 File), a letter of information and consent (S3 File), and a pdf version of PARPHAIT. They were informed about the study, the collection and use of data, and their right to withdraw from the study at any time. If interested, they were asked to confirm their informed consent by e-mail and were contacted by the first author (AE) to schedule the digital interview. Apart from evaluating the content and format of PARPHAIT, data collected also included age, sex, profession, and years of experience.

The invitation was distributed to 80 persons with relevant experience, of which 13 accepted the invitation. Six of the e-mail addresses appeared invalid, 13 invitees declined, while the remaining did not respond or follow up. Among the ones declining, four did however provide written comments in their e-mail. Provided their consent, we also included this written feedback in subsequent analyses.

The mean age of the interviewed sample was 49.7 (range 29–80), and seven (53.8%) were men, and they were heterogeneous with regards to their background, profession, and clinical experience. Sample characteristics are presented in Table 1. To ensure anonymity, data files were given ID numbers (removing participants’ initials and identifiable information in the transcripts). Also, age and sex were not presented for each individual participant. Demographic data were not collected for subjects providing written comments by e-mail.

**Table 1:** Sample characteristics.

ID	Years of experience	Expertise	Profession/role
**ID-1**	10	Sinonasal disease, rhinoplasty, anosmia, parosmia, phantosmia, and taste disorders	ENT^a^ specialist
**ID-2**	11	Anosmia, parosmia, and phantosmia	Patient association representative
**ID-3**	3	Chronic rhinosinusitis, post-viral (including COVID-19) and trauma-related dysfunction, anosmia, parosmia, and phantosmia	ENT specialist and researcher
**ID-4**	20	Chronic rhinosinusitis, anosmia, parosmia, phantosmia, and taste disorders	ENT specialist and researcher
**ID-5**	5	Anosmia, parosmia, and phantosmia	Dental surgeon and researcher
**ID-6**	10	Rhinosinusitis, hyposmia, anosmia, and parosmia	Professor and researcher (Otolaryngology)
**ID-7**	20	Anosmia	Professor (psychological science)
**ID-8**	35	Anosmia, parosmia, and phantosmia	PhD in biology
**ID-9**	10	Normal olfactory function, hyposmia, anosmia, and parosmia	Postdoctor (food science and neuroscience)
**ID-10**	20	Chronic rhinosinusitis, anosmia, parosmia, and phantosmia	ENT specialist
**ID-11**	3	Anosmia, parosmia, and phantosmia	Postdoctor (smell loss)
**ID-12**	40	Anosmia, taste disorders, and trigeminal dysfunction	Medical doctor
**ID-13**	20	Anosmia, parosmia, and phantosmia	Psychologist

^a^Ear, nose, and throat.

### Data collection

Data collection occurred between November 2023 and February 2024. During semi-structured interviews, demographic data and feedback on different aspects of PARPHAIT were collected. Interviews were recorded (audio only) using the Nettskjema-Diktafon mobile app [14], after which audio files were exported and stored in Nettskjema [15]. Saturation of data was considered satisfactory when comments were repeated [16], at which point data collection discontinued. The interview followed one pre-defined interview guide (S2 File), however the order of questions differed somewhat across interviews. Aspects of PARPHAIT included duration and length, relevance and scientific grounding of included items, structure, clarity, definitions/descriptions, response design, scoring, and applicability. Duration and length included the time to complete, the visual perception of length, and the number of items. Relevance and scientific grounding imply the items’ degree of importance, applicability, and support in research. Structure included the distinct sub-scales (loss of smell, phantosmia, and parosmia) and the visual design of these sub-scales. Clarity refers to the formulation of items, how understandable and lengthy they were, and this also applies to the definitions/descriptions presented prior to the items. The response design implies the Likert scale, its number and wording of alternatives, while scoring includes the means of quantifying responses into concrete scores reflecting olfactory function. Finally, applicability refers to PARPHAIT’s user-friendliness and suitability in a clinical setting (e.g., during an ENT specialist consultation). The experts were also invited to address aspects or questions not covered in the interview. All interviews were conducted by one assessor (AE).

### Data analysis

The mean duration (mm:ss) of interviews was 43:26, and ranged from 26:16–57:06. Interviews were transcribed using the AI-service Autotekst [17]. To ensure data were reliably transcribed, text output was reviewed and compared to the recorded audio data by one assessor (AE).

To analyse the data collected, we applied a directed content analysis [18]. Relevant aspects were predefined prior to the interviews, although novel themes not addressed were also expected to arise during the interview and later analyses. Thus, a directed content analysis was considered adequate to synthesise the data. Text was analysed using NVivo 14 [19]. The codes correspond to the aspects in the interview guide, as well as generated novel codes where applicable. As such, the analysis was primarily deductive, although an inductive approach was applied to address novel issues not yet covered in the interview guide or in the predefined codes. To ensure the reliability of data synthesis a sample of three transcripts were also assessed by another assessor (KVM-H). Results were compared, followed by deciding a final set of codes.

The predefined codes included “use in clinical setting, relevance of items” (i.e., questions and aspects that have previously been part of PARPHAIT), “formulations of items”, “introductory text and symptom definitions”, “length”, “potential items” (i.e., questions and aspects that have not previously been part of PARPHAIT), “response design”, “scoring design”, “structure”, and “time frame*”*. The novel codes identified through data analysis were “accessibility” (font, colour, size, etc.), “domain or component”, “patients’ own insight”, “administration”, “control questions”, “quality of life”, “screening questions” and “developing the scale”. These were further sorted into broader codes: “Applicability”, “clarity”, “format”, and “aspects not covered”.

Results were evaluated in light of our previous findings [2], patients’ content validation, the suggested version following EFA [13], and experts’ feedback.

## Results

The quality assessment of PARPHAIT is presented in the order of codes and sub-codes below (also presented and exemplified in S1 Table). The changes applied are summarised in S2 Table.

### Applicability

The “applicability” code contains the sub-codes “administration”, “patients’ own insight”, “relevance of items”, “time frame”, and “use in clinical setting”.

### Administration

The need and suitability of a digital version over delivering it as a paper-pencil questionnaire was emphasised. This was suggested to be favourable in terms of time and cognitive demands spent both in completing and scoring PARPHAIT.

### Patients’ own insight

The participants highlighted the importance of patient’s own insight and meta-knowledge by noting how they may not be aware of their symptoms and distinguish between parosmia and phantosmia.

[…] sometimes if you don’t specifically ask the question to patients, they don’t report the complaint spontaneously. So yes, I think it’s interesting (ID-1).

### Relevance of items

Some questions were considered too specific (e.g., freshly mowed grass), unclear (e.g., dog pile), repetitive (e.g., “I perceive phantom smells …”), and irrelevant (e.g., orthonasal olfaction, due to how patients may be unaware of this and the distinction between ortho- and retronasal olfaction). One suggestion was to focus on environments, such as being at home or at work, more than the difference between retro- and orthonasal olfaction. Regarding neutral phantom smells, this item was considered vague by some, while others regarded it as relevant as patients did not necessarily characterise the phantom smell as pleasant or unpleasant, but rather focused on the change in quality.

Generally, the efforts in assessing phantosmia were acknowledged, as the focus on this phenomenon has received less attention compared to parosmia. Furthermore, the link to emotion and disgust in particular was emphasised, however with a critical view on causal direction. As such, it is unknown whether phantosmia causes the feeling of disgust or if being disgusted provokes phantosmia. Moreover, some people may be more prone to disgust than others, which may also affect their response to the question.

For the parosmia sub-scale, several confirmed the relevance of chocolate, coffee, onion, nuts, meat, and thiol-containing foods. Few had come across rice and milk, however, and considered these irrelevant. Some noted how eating preferences and patterns, allergies, and seasonal and geographical availability impact the relevance of food items.

### Time frame

Regarding the time frame (i.e., past week) some favoured the temporal limitation due to memory recall, while others considered a longer time period as important to better capture symptoms. One argument for a longer time frame was the saliency of qualitative symptoms, leading to a vivid memory of the experience. Symptom fluctuations and the patients’ need to communicate their experiences were also emphasised, irrespective of when these perceptions had taken place. Another argument for extending the time period was the lower likelihood of encountering triggers, especially food triggers, during the course of one week.

### Use in clinical setting

Several considered PARPHAIT a time-efficient tool applicable in clinical practice, depending on the clinical setting, as procedures and patient groups differ across practices. For instance, it could be administered during the waiting time before an appointment in an ENT-practice.

[…] that would take time in conversation. And if the person is clearly parosmic […] they can ask more tailored questions. So that I would see as helpful. It would save them time (ID-13).

Especially the phantosmia sub-scale was of interest, as previous tools focus very little on this symptom. It was considered to provide added value in helping patients understand and cope with their symptoms, and to improve the understanding of underlying mechanisms in the scientific community. Some argued PARPHAIT could not replace the clinical interview but could work as a complementary tool. Given the other tools currently used in practice, some considered its added value limited, noted the unreliability of self-reported symptoms and thought the burden of completing PARPHAIT was too high in its current form.

### Clarity

The “clarity” code contains the sub-codes “formulation of items”, “introductory text and symptom definitions”, “accessibility”, and “domain or component”.

### Formulations of items

Item formulations were considered clear by most, although possible improvements were suggested. Specific and ambiguous items (e.g., “dog pile” and “nuts”) were clarified (i.e., “bathroom odours” and “peanuts”). Moreover, one participant pointed to the issue of knowing what part of the statement one is responding to. The use of words like “perceive”, “detect”, and “recognise” was reported as confusing since these concepts are not interchangeable processes. Instead of using “perceive” some suggested wording the question as “I do not smell…” instead. Similarly, for the phantosmia sub-scale, “I perceive…” should be changed to “I have phantom smells…”. Some did not consider item 16 to be clear, as they did not understand what was meant by a “neutral” phantom smell. The formulations of questions were further regarded as repetitive, and some suggested having a sentence at the very top of the sub-scale: e.g., “My phantom smells…” followed by choosing the alternatives that apply (e.g., “were pleasant”, “happened less than once a week”), with emphasis on the past versus present tense. For the items on valence, one suggestion was to combine these into one item: e.g., “When I perceive a phantom smell, it is typically…” followed by the alternatives “foul”, “neutral”, and “pleasant”. Regarding causal direction the item on disgust could be rephrased as “When I am disgusted, I perceive phantom smells more often” to clearly define that the emotional state causes the phantom smell and not vice versa. Food triggers for parosmia were unclear as to whether the measured outcome was the changed perception of the food items or their triggering effect on other odours. Also, frequency questions could be improved (e.g., “at least once every day”), and the environmental aspect was rephrased as: “I perceive phantom smells more often indoors than when I am outdoors”.

### Introductory text and symptom definitions

Most participants reported the text to be clear and comprehensible, however with suggestions to possible changes and improvements. For instance, using fewer syllables and a plain language was emphasised. One suggestion was to reduce the number of items in the quantitative sub-scale and use some of these as illustrative examples in the introductory text instead. Others suggested enhancing memory recall by asking the patient to picture concrete situations. Some believed patients would know what symptoms implied without further elaboration, while most considered examples important to ensure valid and reliable responses.

[…] you want to put people in the condition to really understanding what you’re trying to measure, because we’re not used to making these distinctions. It’s not that you can rely on people’s self-knowledge about parosmia (ID-13).

The explanation of how “changed and different(ly) here does not include symptoms of a reduced or lost sense of smell” was considered unclear and should be reformulated.

### Accessibility

Considering the appearance of the questionnaire, one suggestion was to increase the font size and use different colours to clarify aspects and response options. Another was to use icons instead of text, and to present the sub-scales on separate pages, if presented in a paper-pencil version.

### Domain or component

The different meanings of the words “perceive”, “recognise”, and “do not mind” were emphasised. It was advised to better clarify what was being measured, as these terms translate into odour awareness, identification, and hedonicity, respectively.

### Format

The “format” code contains the sub-codes “length”, “developing the scale”, “response design”, “scoring design”, and “structure*”*.

### Length

Most participants considered the length adequate relative to other olfactory questionnaires. The estimated time to complete ranged from less than five to about 15 minutes. Suggestions were made about shortening the questionnaire (i.e., one page) to enhance its applicability. Using a triage system and combining some items could be ways of achieving this.

### Developing the scale

One suggested reference for scale development was the disgust scale by Haidt and colleagues [20], while another emphasised the need to further test the scale on a patient population to assess its applicability. Regarding the food triggers for parosmia, it was noted how disgust sensitivity differs across patients, and that a more thorough assessment of this could aid in choosing the relevant trigger items.

### Response design

To most participants the response design was considered adequate, easy to understand, and clinically applicable, while some had suggestions for improvement. For instance, the “not applicable” option may be confused with “disagree” and “neither agree nor disagree”, which would make interpretation challenging. While including “not applicable” could ensure completion of items, some thought it could be an easy way out without really considering the question. Removing the “not applicable” and “neither agree nor disagree” was noted as options, depending on whether the aim is to ensure discriminative ability or avoiding choosing the neutral (middle) option. One suggestion was to place “agree” on the right-hand side, as this was considered more intuitive.

The use of one response design for all questions was reported by some to be inadequate, as some aspects are measured in binary terms (e.g., triggers), while others are scored on a continuum (e.g., frequency). Some suggested using a visual analogue scale (VAS) or a multiple-choice design.

Regarding food triggers, some favoured the idea of a free text entry, while others did not because of how people differ in how descriptive they are and how much they remember. As food triggers could be scored in binary terms, a Likert scale would not contribute to capturing any additional information. One suggestion was to measure the number of triggers with an interval response design: “0-5”, “5-10”, “more than 10”, etc. Another was to collect triggers and non-triggers, however more as a means of understanding what triggers were the most applicable. The confirmed relevance of these items would then increase the validity in the further development of the scale.

### Scoring design

Participants were generally positive to the idea of having both sub-scores and a total score. A standardised, validated tool was emphasised, as was the assessment of a minimally important difference, and a linear scale (with scores from 0 to 5) over a logarithmic design. A VAS or slider option where item scores were summarised and averaged for each item was another idea. Some considered the mere addition of single item scores inadequate, while others reported that summing up scores were normal procedure in clinical practices. However, one suggested lower scores would better distinguish those with symptoms from those without, and preferred subtracting from the score whenever disagreeing with a statement. Some considered weighted scores to be useful, as the psychophysical distance between one response and the other may not be equal. Moreover, aspects should be considered in combination when calculating the score.

PARPHAIT was considered well-designed for determining cut-off scores, however with some challenges. For instance, which items are more important – the inability to smell flowers or a damp cellar? Depending on the distribution it would also be important to consider those responding “somewhat agree” (and not only “agree”) since some may not use the extremes of the scale when they respond. One suggestion was to categorise responses into two categories: if affected by 30 different food items, one would have “broad” parosmia, while less food items correspond to a “narrow” type. Some thought the number of triggers could be a good indicator of parosmia severity, where pleasant odours should have a lower impact than unpleasant ones. However, another noted that even if the experience of an odour is pleasant, it may not be a positive experience if it occurs constantly and instead of the odour normally perceived. Food triggers could be scored a point each, but this idea was questionable because of how it would diverge from the overall, fixed scoring design.

### Structure

Most were positive to including three sub-scales as part of one holistic measure of olfactory dysfunction. PARPHAIT was reported to be nicely presented, and a well-suited format for time-effective means of measuring symptoms in a clinical context. Some were not convinced that anosmia, hyposmia, parosmia, and phantosmia are mutually exclusive, which could complicate the diagnosis of specific symptoms. Others believed one phenomenon is likely to exclude another and did not consider this an issue. Sub-scales should resemble each other in terms of number of items and the aspects covered, as these were currently presented somewhat randomly.

### Aspects not covered

This code contains the sub-codes “potential items”, “control questions”, “quality of life”, and “screening questions”.

### Potential items

Some mentioned including more positive odours (e.g., food and fragrances). Social relationships, risk situations (e.g., recognising a fire), one’s own (and not just others’) body odour and perspiration, triggering factors like seasons, physical activity, smoking, comorbidities, environments devoid of olfactory stimuli, toothpaste, cucumber, roasted food, spoiled food, and non-triggering items were mentioned. Moreover, the positive change of odours, such as not being bothered by unpleasant bathroom smells, could be relevant to include, and so could fluctuations, duration, and severity of parosmia and phantosmia. Regarding phantosmia, one participant mentioned the identification of the phenomenon through blocking nasal airflow. If odour sensations persisted, this was likely phantosmia. As such, this could be included as a description or an item in the questionnaire.

### Control questions

One suggestion was the aforementioned anchors that generally would not provoke qualitative symptoms. Another concerned the inclusion of control questions to ensure responses were genuine and consistent.

### Quality of life

Several mentioned the relevance of addressing quality of life, and the difference between being annoyed and distressed by symptoms, of which the latter indicates symptom severity to a greater extent.

Coffee doesn’t smell like it used to, but it doesn’t smell disgusting to them. That is not captured, or the difference between them and the people who are so parosmic they can’t drink coffee is not captured in these questionnaires […] That’s an impact on quality of life and something that you want to do something about.” (ID-10).

### Screening questions

Screening questions prior to the sub-scales was advised to reduce demands, increase question relevance, and also to account for confounding variables. This could work as a triage system, so that the following questions apply to the right patient. Regarding confounding variables, migraine, antihypertensive medication, chemotherapy, smoking, and other aetiologies like congenital anosmia and head trauma were noted as relevant variables.

## Discussion

### Principal findings

Experts’ evaluation of PARPHAIT resulted in both confirming and challenging the current content and design.

Changes were applied to formulations of items and introductory text for improved clarity, readability, and resemblance between sub-scales. Some words (e.g., “dog pile”) were considered ambiguous and unclear, both in the current study and during the content validation in patients [13]. These were removed and replaced by more common words describing similar odours (e.g., “bathroom odours”, which was also used in previous research [5]). We reduced the length of sentences, aimed for syntax resemblance between texts, and presented examples using odours most people would be familiar with.

The time frame was extended from the “past week” to “past two weeks” to better capture symptoms and include relevant patients. The time frame differs across questionnaires that is currently used in practice and research. For instance, the Olfactory Dysfunction Outcomes Rating developed by Lee and colleagues [10] has a time frame of thirty days, while another, the 22-item Sinonasal Outcome Test [21] accounts for the past two weeks. Given the increased likelihood of recall bias as time passes, this may reduce the reliability of responses. Therefore, extending the time frame by one week was considered adequate.

To reduce the length, some items were combined. This necessitated the inclusion of several concepts in one single question, potentially limiting internal validity to some degree. For instance, the item “… pungent odours like sweat, “bathroom odours”, or mold” includes three unpleasant, yet different odours. Furthermore, “bathroom odours” includes more than one given odour, and the experience of such odours may also vary, both in chemical composition and perception. When the item includes several examples, it is unknown which of the odours apply. Specific examples may also introduce bias, leading to not considering odours beyond the examples [22]. However, the compression of items was done to reduce cognitive demands of completing PARPHAIT and to increase the applicability of the item. Following the combination of items, the phantosmia sub-scale was reduced to 12, while the parosmia sub-scale was increased to 18 items. In sum, the current version of PARPHAIT (S4 File) has 32 items.

The response design was made more intuitive by placing “agree” on the right-hand side and “disagree” on the left. This may also reduce bias with regards to the direction and placement of responses [23], although such effects may not necessarily occur [24]. One suggestion was to alter response options according to the nature of the question (e.g., “every day” etc. for frequency questions). This was not changed, however, due to aiming for a clear, uniform design. Having different scales for distinct aspects would increase the cognitive demand as the patient would have to adapt to each new aspect. Compared to a VAS design, the Likert scale appears more suitable because of how response options are defined and less prone to subjective interpretation, both in providing and scoring responses. Moreover, the reliability may increase with a six-item response design [25]. The Questionnaire of Olfactory Disorders [26] has a scale of four options, while the Self-reported Mini Olfactory Questionnaire [27] only includes a binary response design, giving the participant the options of yes or no. As such, PARPHAIT offers an improvement in terms of capturing reliable responses.

Inspired by findings in the systematic review, we included a range of aspects of olfactory dysfunction. Such aspects have previously been addressed, both relative to COVID-19 [5] and other aetiologies (like head trauma and toxic exposure [28]) but are not scored and standardised. One recent contribution that ticks these boxes is the Taste and Smell Tool for Evaluation Questionnaire [11]. Despite its promising ability of capturing olfactory symptoms, it assesses parosmia and phantosmia each with only one question and combines the scores into one “chemosensory distortion” score. This score also includes two questions on taste alteration. Moreover, the questionnaire does not include clear definitions as to what “distortions” imply and whether or not it may also involve loss of smell. PARPHAIT aims to provide clear definitions and measure parosmia and phantosmia as separate constructs.

We decided to include cucumber and toothpaste to the parosmia sub-scale, and smoke to the phantosmia sub-scale, as these were frequently mentioned during interviews. The added description “cigarette or campfire smoke” was provided to clarify that smoke was measured as a general phenomenon. While specific items for each type would increase validity, fewer items and higher feasibility were prioritised. Hörberg and colleagues [29] identified triggers of parosmia, of which the most common ones were generally unpleasant odours (e.g., excrement). Despite this, previous research (e.g., [5]) tend to focus on pleasant trigger odours, as does PARPHAIT. Hörberg and colleagues suggest pleasant odours may have had a greater focus in research due to how negative changes in odour perception affects quality of life to a greater extent than positive changes. PARPHAIT has focused largely on food-related triggers, and more unpleasant items could be relevant for inclusion. Some items previously removed after EFA were reintroduced (i.e., valence and quality). The distinction between an odour’s pleasantness and quality is important to note as these are not interchangeable. Experiencing an odour differently does not necessarily imply a change in both pleasantness and quality; some may only perceive it as more or less pleasant, while others characterise it as different without it being changed in its valence. Therefore, treating these independently and making clear the distinction to patients will increase PARPHAIT’s ability to capture and characterise olfactory symptoms.

Another suggestion was to include screening questions as part of a triage system. While this would reduce cognitive demands and ease the completion of PARPHAIT, it would also overlook one of the main purposes of the tool: to capture specific symptoms that would not necessarily be identified if only asked about olfactory function in general. One cannot assume that all patients are aware of specific symptoms, that they know their condition well, or notice the relationship between a trigger and a symptom. We could also have included quality of life questions and screen for comorbidity, however the focus of PARPHAIT is to capture specific symptoms. Quality of life is already measured using other questionnaires (e.g., [10,11,30]), and comorbidities should be considered part of standard procedures [31]. Finally, control questions that ensure responses are genuine (such as those used in the Questionnaire of Olfactory Disorders [26]) were not included in the current version. These serve an important function with regards to reliability but was not prioritised due to aiming for a short and feasible questionnaire.

### Strengths and weaknesses

One of the strengths of our study is the inclusion of items specific to the loss of smell, parosmia, and phantosmia. Oftentimes, the latter two are measured or reported as one construct, although they are different both as phenomena and in terms of their impact. PARPHAIT addresses this issue by clearly describing and capturing aspects of parosmia and phantosmia, and puts emphasis on phantosmia, which has previously not been the focus of attention.

The study also has its limitations, one of which concerns the sample size and data saturation. According to Saunders et al. [16] data collection is satisfactory when data is repeated, which took place after 13 interviews in our study. A higher number of participants could improve the robustness of our findings. However, experts were strategically selected based on their competence and we believe reliable data were obtained through the two-phased study design.

Bias may also have been introduced during interviews. Given the novelty of PARPHAIT, some requested more information. The time spent on reviewing PARPHAIT also varied across participants, and the interviewer’s responses to feedback may have influenced the engagement, reflection, and the answers given.

### Implications for the scientific and clinical community

By introducing PARPHAIT to the scientific and clinical community, we can increase our knowledge about how symptoms are characterised and what provokes them. This may improve the methodology of research studies focused on qualitative olfactory dysfunction, as symptoms hitherto often have been measured and reported ambiguously. As such, the validity of studies may improve, and PARPHAIT can ease demands in clinical practice by working as an assessment tool prior to or during clinical consultations. It may contribute to valid diagnoses in combination with psychophysical tests [32], which offers a more objective measure of symptoms, although perhaps being more demanding in terms of cost and time resources. Furthermore, answering specific questions about patients’ condition could bring to mind experiences not otherwise recalled unless specifically asked about it. As such, it may provide patients with a deeper insight and understanding of their own condition, which in turn could also aid the communication between the patient and clinician.

### Future research implications

Future studies may involve the assessment of the most recent version of PARPHAIT by distributing it to a (larger) patient group. This study should also include a control group for comparison and ensuring that the questionnaire is able to distinguish patients from controls without symptoms. Following this process, a confirmatory factor analysis could aid in determining the inclusion of items and to assess whether the current factor structure is adequate. It would be useful to include additional aspects, to test different response designs (e.g., VAS) and administration (paper-pencil vs. digital), followed by re-assessing factor structure, reliability, and feasibility in a clinical context.

Finally, a scoring protocol would need to be developed and refined based on the data collected. This could involve a theory-driven method, where different strategies are worked out and tested, or rely more on statistical procedures like item response theory.

## Conclusion

The purpose of this study was to evaluate the content validation of PARPHAIT in an olfactory expert group, with the aim of improving its quality and content validity through guided refinement and adaptation. PARPHAIT was found to have potential as a useful tool in clinical practice, provided the reformulation of items, instructions, and definitions, extension of the time frame, combination of items, re-ordering and re-structuring sub-scales, and the inclusion and reintroduction of items. Developing a novel, robust tool requires time and pragmatic methods of assessing its quality and applicability, and more work still remains in order to settle on a version that can be employed in a clinical context.

## Supporting information

S1 File PARPHAIT distributed to experts. The version of PARPHAIT as distributed to the experts involved in the study.
(DOCX)

S2 File Interview guide. The pre-defined questions distributed prior to and used during individual interviews.
(DOCX)

S3 File Information and letter of consent. Letter of consent distributed to experts involved in the study.
(DOCX)

S4 File Current version of PARPHAIT. The current version of PARPHAIT after patient content validation, exploratory factor analysis (EFA), and expert content validation.
(PDF)

S1 Table Codes, sub-codes, and illustrative quotes. (DOCX)

S2 Table Changes applied to PARPHAIT after patient content validation, exploratory factor analysis (EFA) a, and expert content validation. (PDF)

## References

[1] Hummel T, Whitcroft KL, Andrews P, Altundag A, Cinghi C, Costanzo RM, et al. Position paper on olfactory dysfunction. Rhinology. 2016;56(1):1–30. doi: 10.4193/Rhino16.248 28623665

[2] Espetvedt A, Wiig S, Myrnes-Hansen KV, Brønnick KK. The assessment of qualitative olfactory dysfunction in COVID-19 patients: a systematic review of tools and their content validity. Front Psychol. 2023;14:1190994. doi: 10.3389/fpsyg.2023.1190994 37408960 PMC10319418

[3] Chu M, Gopikrishna D, Rocke J, Kumar BN. Implementing a COVID-19 specialist smell clinic: experience at the Wrightington, Wigan and Leigh Teaching Hospitals (WWL), NHS Foundation Trust, United Kingdom. Med J Malaysia. 2021;76(Suppl 4):9–13. 34558550

[4] Bussière N, Mei J, Lévesque-Boissonneault C, Blais M, Carazo S, Gros-Louis F, et al. Chemosensory Dysfunctions Induced by COVID-19 Can Persist up to 7 Months: A Study of Over 700 Healthcare Workers. Chem Senses. 2021;46:bjab038. doi: 10.1093/chemse/bjab038 34423831 PMC8499810

[5] Parker JK, Methven L, Pellegrino R, Smith BC, Gane S, Kelly CE. Emerging Pattern of Post-COVID-19 Parosmia and Its Effect on Food Perception. Foods. 2022;11(7). 10.3390/foods11070967 PMC8997629 35407054

[6] Weiss JJ, Attuquayefio TN, White EB, Li F, Herz RS, White TL, et al. Tracking smell loss to identify healthcare workers with SARS-CoV-2 infection. PLoS One. 2021;16(3):e0248025. doi: 10.1371/journal.pone.0248025 33657167 PMC7928484

[7] Otte MS, Haehner A, Bork ML, Klussmann JP, Luers JC, Hummel T. Impact of COVID-19-Mediated Olfactory Loss on Quality of Life. ORL J Otorhinolaryngol Relat Spec. 2023;85(1):1–6. 35413715 10.1159/000523893 PMC9148912

[8] Klein H, Asseo K, Karni N, Benjamini Y, Nir-Paz R, Muszkat M. Onset, duration, and persistence of taste and smell changes and other COVID-19 symptoms: longitudinal study in Israeli patients. Clin Microbiol Infect. 2021;27(5):769–74. 33607252 10.1016/j.cmi.2021.02.008 PMC7884919

[9] Makaronidis J, Firman C, Magee CG, Mok J, Balogun N, Lechner M, et al. Distorted chemosensory perception and female sex associate with persistent smell and/or taste loss in people with SARS-CoV-2 antibodies: a community based cohort study investigating clinical course and resolution of acute smell and/or taste loss in people with and without SARS-CoV-2 antibodies in London, UK. BMC Infect Dis. 2021;21(1):221. doi: 10.1186/s12879-021-05927-w 33632171 PMC7905973

[10] Lee JJ, Mahadev A, Kallogjeri D, Peterson AM, Gupta S, Khan AM, et al. Development and Psychometric Validation of the Olfactory Dysfunction Outcomes Rating. JAMA Otolaryngol Head Neck Surg. 2022;148(12):1132–9. doi: 10.1001/jamaoto.2022.3299 36264557 PMC9585455

[11] Niklassen AS, Christensen KB, Fjaeldstad AW, Ovesen T. Development and Psychometric Validation of the Taste And Smell Tool for Evaluation (TASTE) Questionnaire. JAMA Otolaryngol Head Neck Surg. 2022;148(12):1164–72. doi: 10.1001/jamaoto.2022.3392 36326741 PMC9634595

[12] Lerner DK, Garvey KL, Arrighi-Allisan AE, Filimonov A, Filip P, Shah J. Clinical Features of Parosmia Associated With COVID-19 Infection. Laryngoscope. 2022;132(3):633–9. 34870334 10.1002/lary.29982 PMC9015517

[13] Espetvedt A, Brønnick KK, Wiig S, Myrnes-Hansen KV, Lungu DA. Capturing qualitative olfactory dysfunction with PARPHAIT: the parosmia, phantosmia, and anosmia test. Rhinology Online. 2024;7:39–65.

[14] Universitetet i Oslo. Nettskjema-diktafon [Mobile app]. 2017.

[15] Universitetet i Oslo. Nettskjema. 2012. Available from: www.nettskjema.no/user/form

[16] Saunders B, Sim J, Kingstone T, Baker S, Waterfield J, Bartlam B, et al. Saturation in qualitative research: exploring its conceptualization and operationalization. Qual Quant. 2018;52(4):1893–907. doi: 10.1007/s11135-017-0574-8 29937585 PMC5993836

[17] Universitetet i Oslo. Autotekst. 2023. Available from: www.autotekst.uis.no

[18] Hsieh HF, Shannon SE. Three Approaches to Qualitative Content Analysis. Qual Health Res. 2005;15(9):1277–88. 16204405 10.1177/1049732305276687

[19] Lumivero. NVivo (Version 14). 2024.

[20] Haidt J, McCauley C, Rozin P. Individual differences in sensitivity to disgust: A scale sampling seven domains of disgust elicitors. Pers Individ Dif. 1994;16:701–13.

[21] Hopkins C, Gillett S, Slack R, Lund VJ, Browne JP. Psychometric validity of the 22-item Sinonasal Outcome Test. Clin Otolaryngol. 2009;34(5):447–54. doi: 10.1111/j.1749-4486.2009.01995.x 19793277

[22] Choi BCK, Pak AWP. A catalog of biases in questionnaires. Prev Chronic Dis. 2005;2(1):A13. 15670466 PMC1323316

[23] Friedman HH, Herksovitz PJ, Pollack S. Biasing effects of scale-checking styles on responses to a Likert scale. In: Proceedings of the American Statistical Association Annual Conference: Survey Research Methods, 1993.

[24] Weng L-J, Cheng C-P. Effects of Response Order on Likert-Type Scales. Educational and Psychological Measurement. 2000;60(6):908–24. doi: 10.1177/00131640021970989

[25] Simms LJ, Zelazny K, Williams TF, Bernstein L. Does the number of response options matter? Psychometric perspectives using personality questionnaire data. Psychol Assess. 2019;31(4):557–66. doi: 10.1037/pas0000648 30869956

[26] Frasnelli J, Hummel T. Olfactory dysfunction and daily life. Eur Arch Otorhinolaryngol. 2005;262(3):231–5. doi: 10.1007/s00405-004-0796-y 15133691

[27] Zou L-Q, Linden L, Cuevas M, Metasch M-L, Welge-Lüssen A, Hähner A, et al. Self-reported mini olfactory questionnaire (Self-MOQ): A simple and useful measurement for the screening of olfactory dysfunction. Laryngoscope. 2020;130(12):E786–90. doi: 10.1002/lary.28419 31747076

[28] Nordin S, Brämerson A, Murphy C, Bende M. A Scandinavian adaptation of the Multi-Clinic Smell and Taste Questionnaire: evaluation of questions about olfaction. Acta Otolaryngol. 2003;123(4):536–42. doi: 10.1080/00016480310001411 12809108

[29] Hörberg T, Sekine R, Overbeck C, Hummel T, Olofsson JK. A parosmia severity index based on word-classification predicts olfactory abilities and impairment. Eur Arch Otorhinolaryngol. 2023;280(8):3695–706. doi: 10.1007/s00405-023-07893-2 36906652 PMC10008075

[30] Zou L, Haehner A, Menzel S, Gunder N, Hummel T. Reliability and validity of a brief version of the Questionnaire of Olfactory Disorders (brief QOD) in patients with olfactory dysfunction. Rhinology. 2021;60. 10.4193/Rhin21.059 34874020

[31] Philpott C, Kumaresan K, Fjaeldstad AW, Macchi A, Monti G, Frasnelli J, et al. Developing a core outcome set for clinical trials in olfactory disorders: a COMET initiative. Rhinology. 2023;61(4):312–9. doi: 10.4193/Rhin22.116 37243690

[32] Holy R, Kalfert D, Vasina L, Vorobiov O, Dytrych P, Janouskova K, et al. Olfactory event-related potentials (OERPs) and trigeminal event-related potentials (TERPs) in subjects after Covid-19 infection: single-center prospective study. J Appl Biomed. 2024;22(3):149–54. doi: 10.32725/jab.2024.020 39434512

