# Supplementary material for: Experts’ content validation of the parosmia, phantosmia, and anosmia test (PARPHAIT): A qualitative study
Source: PLoS One. 2025 Aug 5;20(8):e0329108. doi: 10.1371/journal.pone.0329108 (PMC12324124; doi:10.1371/journal.pone.0329108)
Supplement: S1 File — The version of PARPHAIT as distributed to the experts involved in the study. (DOCX) [file pone.0329108.s001.docx]

S1 File. PARPHAIT distributed to experts.

Below are statements about how you experience your own sense of smell.

On a scale from agree to disagree, how do the following statements apply to you during the past week? If the statement does not apply to you, if you are not sure, or if you are unfamiliar with the odours, please select the "not applicable" option.

|  |  | Not  applicable | Agree | Somewhat  agree | Neither  agree nor  disagree | Somewhat  disagree | Disagree |
| --- | --- | --- | --- | --- | --- | --- | --- |
| 1 | I like to look around the flower shop, but I cannot smell anything | □ | □ | □ | □ | □ | □ |
| 2 | I do not mind the bad smell in some public toilets | □ | □ | □ | □ | □ | □ |
| 3 | I do not recognise the smell of freshly mowed grass | □ | □ | □ | □ | □ | □ |
| 4 | It may happen that I do not notice it when I step into a dog pile | □ | □ | □ | □ | □ | □ |
| 5 | I do not perceive the musty odour in a damp cellar | □ | □ | □ | □ | □ | □ |
| 6 | I do not smell the perspiration of sweaty people | □ | □ | □ | □ | □ | □ |

Below are statements about perceiving odours that other people around you do not experience and that has no apparent odour source. In the statements such odours are referred to as "phantom smells".

On a scale from agree to disagree, how do the following statements apply to you during the past week? If the statement does not apply to you, if you are not sure, or if you are unfamiliar with the odours, please select the "not applicable" option.

|  |  | Not  applicable | Agree | Somewhat  agree | Neither  agree nor  disagree | Somewhat  disagree | Disagree |
| --- | --- | --- | --- | --- | --- | --- | --- |
| 7 | I perceive phantom smells more often when I eat | □ | □ | □ | □ | □ | □ |
| 8 | I perceive phantom smells more often when I am disgusted | □ | □ | □ | □ | □ | □ |
| 9 | I perceive phantom smells about once a day | □ | □ | □ | □ | □ | □ |
| 10 | I perceive phantom smells intensely | □ | □ | □ | □ | □ | □ |

|  |  | Not  applicable | Agree | Somewhat  agree | Neither  agree nor  disagree | Somewhat  disagree | Disagree |
| --- | --- | --- | --- | --- | --- | --- | --- |
| 11 | I perceive phantom smells more often when breathing through my nose | □ | □ | □ | □ | □ | □ |
| 12 | I perceive phantom smells' strength like other odours | □ | □ | □ | □ | □ | □ |
| 13 | I perceive phantom smells about once a week | □ | □ | □ | □ | □ | □ |
| 14 | I perceive phantom smells more often when I am indoors | □ | □ | □ | □ | □ | □ |
| 15 | I perceive pleasant phantom smells | □ | □ | □ | □ | □ | □ |
| 16 | I perceive neutral phantom smells | □ | □ | □ | □ | □ | □ |
| 17 | I perceive phantom smells less than once a week | □ | □ | □ | □ | □ | □ |
| 18 | I perceive unpleasant phantom smells | □ | □ | □ | □ | □ | □ |
| 19 | I perceive phantom smells only vaguely | □ | □ | □ | □ | □ | □ |

Below are statements about perceiving odours differently from how they usually smell like. By this we mean a change in the character of an odour and/or the pleasantness of that odour. For instance, coffee may now smell like garbage, and smells foul when it perhaps previously smelled pleasant to you. It can also be that something that previously smelled unpleasant now smells more pleasant. Note that changed and different(ly) here does not include symptoms of a reduced or lost sense of smell.

On a scale from agree to disagree, how do the following statements apply to you during the past week? If the statement does not apply to you, if you are not sure, or if you are unfamiliar with the odours, please select the "not applicable" option.

|  |  | Not  applicable | Agree | Somewhat  agree | Neither  agree nor  disagree | Somewhat  disagree | Disagree |
| --- | --- | --- | --- | --- | --- | --- | --- |
| 20 | I perceive odours differently more often when I eat or prepare nuts | □ | □ | □ | □ | □ | □ |
| 21 | I perceive odours differently more often when I eat or prepare rice | □ | □ | □ | □ | □ | □ |

|  |  | Not  applicable | Agree | Somewhat  agree | Neither  agree nor  disagree | Somewhat  disagree | Disagree |
| --- | --- | --- | --- | --- | --- | --- | --- |
| 22 | I perceive odours differently more often when I eat or prepare fish | □ | □ | □ | □ | □ | □ |
| 23 | I perceive odours differently more often when I eat or prepare tomato | □ | □ | □ | □ | □ | □ |
| 24 | I perceive odours differently more often when I eat or prepare chocolate | □ | □ | □ | □ | □ | □ |
| 25 | I perceive odours differently more often when I eat or prepare meat | □ | □ | □ | □ | □ | □ |
| 26 | I perceive odours differently more often when I eat or prepare lemon | □ | □ | □ | □ | □ | □ |
| 27 | I perceive odours differently more often when I drink or prepare coffee | □ | □ | □ | □ | □ | □ |
| 28 | I perceive odours differently more often when I drink or prepare milk | □ | □ | □ | □ | □ | □ |
| 29 | I perceive odours differently more often when I eat or prepare eggs | □ | □ | □ | □ | □ | □ |
| 30 | I perceive odours differently more often when I eat or prepare melon | □ | □ | □ | □ | □ | □ |
| 31 | I perceive odours differently more often when I eat or prepare onion | □ | □ | □ | □ | □ | □ |
| 32 | I perceive odours differently about once a day | □ | □ | □ | □ | □ | □ |
| 33 | I perceive odours differently about once a week | □ | □ | □ | □ | □ | □ |
| 34 | I perceive odours differently about once an hour | □ | □ | □ | □ | □ | □ |
