# Supplementary material for: Experts’ content validation of the parosmia, phantosmia, and anosmia test (PARPHAIT): A qualitative study
Source: PLoS One. 2025 Aug 5;20(8):e0329108. doi: 10.1371/journal.pone.0329108 (PMC12324124; doi:10.1371/journal.pone.0329108)
Supplement: S2 File — The pre-defined questions distributed prior to and used during individual interviews. (DOCX) [file pone.0329108.s002.docx]

**S2 File. Interview guide**

The interviews will follow a structured format. However, depending on the answers given, additional questions may follow to allow for further exploration within a given topic. Apart from the first three questions covering participant characteristics, the questions asked will concern only PARPHAIT and its content.

#### **Participant characteristics**

1. What is your age?
2. What is your profession/role? What does it involve?
3. How long have you been working/interested in the field of olfaction?

#### **Duration**

1. How did you experience the length and duration of the scale?
2. How long did it take?

#### **Relevance**

1. What are your thoughts on the relevance of items?
2. How would you consider the scientific grounding of included aspects?
3. Are there any aspects that we have yet included that you think would be relevant?

#### **Structure**

*It seems difficult to design a scale that captures all three symptoms (anosmia, phantosmia, and parosmia) that also still remain relevant to all. As such, many patients may experience phantosmia, but no other symptoms, while others have parosmia only.*

1. What are your thoughts on including all three aspects in the scale?
2. Could this be improved? How?

#### **Clarity**

1. How would you evaluate the clarity of questions?
2. Did you find any particular question difficult to understand or read?
3. How would you rephrase the(se) question(s)?
4. Overall, how would you rate the clarity of the scale from 1 to 5 (where 5 is “very clear”)?

#### **Definitions**

1. How would you evaluate the clarity of the symptom definitions and descriptions given before the questions?
2. Would you have described this differently? How?

#### **Response design**

1. How would you consider the response design?
2. Do you think these should have been worded differently?

#### **Scoring**

*As a starting point, we suggest the following scoring design for the PARPAIT: “not applicable”- 0, “agree”- 3, “somewhat agree”- 2, “neither agree nor disagree” - 0, “somewhat disagree” - 1, and “disagree” - 0.*

1. How would you consider this way of scoring responses?
2. Would you recommend applying a weighted score to the items? Why/why not?
3. How should we determine cut-off scores?
4. How should we determine labels corresponding to final scores?

*We want to give sub-scores for each phenomenon as well as a total score for the scale in its entirety.*

1. What are your thoughts on this?

*For the trigger questions, these may be relevant for some, but not for others. Also, there may be triggers that are not covered here. We could add a free-text entry where triggers can be noted, and perhaps scored depending on the number of triggers, their intensity, or duration?*

1. Do you have any suggestions to how this could be done?

#### **Use in clinical setting**

1. How would you consider the degree of user-friendliness and applicability related to the work/role you have?

***Other***

**Q26** Is there anything you would like to add that questions did not cover?
