# Supplementary material for: Experts’ content validation of the parosmia, phantosmia, and anosmia test (PARPHAIT): A qualitative study
Source: PLoS One. 2025 Aug 5;20(8):e0329108. doi: 10.1371/journal.pone.0329108 (PMC12324124; doi:10.1371/journal.pone.0329108)
Supplement: S3 File — Letter of consent distributed to experts involved in the study. (DOCX) [file pone.0329108.s003.docx]

## **S3 File. Content evaluation of the parosmia, phantosmia, and anosmia test (PARPHAIT)**

Letter of informed consent

We would like to invite you to participate in our study, where the aim is to assess the content and quality of a novel questionnaire. The parosmia, phantosmia, and anosmia test (PARPHAIT) is developed to measure both quantitative and qualitative symptoms of olfactory dysfunction, with primary focus on the latter.

PARPHAIT assesses the loss of smell, parosmia, and phantosmia through three sub-scales. We have previously evaluated the face validity of items in a COVID-19 population who experience such olfactory symptoms. Now, we want to evaluate the adequacy of PARPHAIT in a panel of experts to ensure the quality of the content and format.

**What does participation involve?**

The study involves reviewing the PARPHAIT and participating in a digital interview (using Zoom or Teams).

We first ask you to go through PARPHAIT and to consider its length, duration, relevance, structure, clarity, definitions, response design, scoring, and applicability in practice. Along with PARPHAIT, we provide you with the interview guide in which these aspects are covered. The review of PARPHAIT will take approximately 10 minutes, and the interview will take 45 to 60 minutes.

Should you be interested to participate in a digital interview, we ask you to confirm this by e-mail to annelin.espetvedt@uis.no. You will be contacted shortly by e-mail to schedule a meeting, to which you will receive an e-mail with a link.

Participation is voluntary. Should you wish not to participate or withdraw from the study, you can choose to do so at any time without giving a reason.

**How do we store and use your data?**

During the interview, we will collect the following data: age, sex, profession/role, years of experience within the field of olfaction, and your feedback on PARPHAIT. Personal data will not be analysed or used further in the study, and are only used during data collection. Age, sex, profession/role, and years of experience within the field of olfaction will only be used to describe the population sample.

To enable subsequent analyses, the interview will be recorded (audio only) using the Nettskjema-diktafon mobile app. Data will be encrypted and securely stored in Services for Sensitive Data.

We treat the data collected confidentially and in accordance with the data protection regulations. The data will only be accessed by the research group involved in this project, consisting of the PhD-student Annelin Espetvedt, the main supervisor Daniel A. Lungu, and two co-supervisors Siri Wiig and Kai Victor Myrnes-Hansen, all affiliated with the University of Stavanger in Norway.

It will not be possible to tie published results to your personal data. Data will only be used for the purposes of the research project and will be stored securely until October 2025. After this, all data will be deleted. Provided that you can be identified in the data, you are entitled to access your data, have it rectified or deleted, request a copy, and submit a complaint about the data processing. In this regard, you can contact the Data Protection Officer at personvernombud@uis.no.

Sikt, the Norwegian Agency for Shared Services in Education and Research, has approved the collection, processing, and storage of personal data for this research project (reference ID 282584, approved 31.10.2023).

**Do you have any questions?**

Should you have any questions regarding the project, please contact PhD-student Annelin Espetvedt (annelin.espetvedt@uis.no) or the main supervisor Daniel A. Lungu (daniel.a.lungu@uis.no).
