# Supplementary material for: Experts’ content validation of the parosmia, phantosmia, and anosmia test (PARPHAIT): A qualitative study
Source: PLoS One. 2025 Aug 5;20(8):e0329108. doi: 10.1371/journal.pone.0329108 (PMC12324124; doi:10.1371/journal.pone.0329108)
Supplement: S4 File — The current version of PARPHAIT after patient content validation, exploratory factor analysis (EFA), and expert content validation. (PDF) [file pone.0329108.s004.pdf]

**S4 File. The Parosmia, phantosmia, and anosmia test (PARPHAIT)**

We are interested in your sense of smell. During the **past two weeks**, how much do you agree with the following statements?
If the statement does not apply to you, if you are unsure, or if you are unfamiliar with the odours, please select the "not applicable" option.

| | I could not smell … | Not applicable | Disagree | Somewhat disagree | Neither agree nor disagree | Somewhat agree | Agree |
|---|---|---|---|---|---|---|---|
| 1 | … pleasant odours like flowers or freshly mowed grass | ◻ | ◻ | ◻ | ◻ | ◻ | ◻ |
| 2 | … pungent odours like sweat, "bathroom odours", or mold | ◻ | ◻ | ◻ | ◻ | ◻ | ◻ |

We are interested in the experience of odours that have no apparent odour source, so-called "phantom smells". For example, you may smell cigarette smoke when no-one around you is smoking. During the **past two weeks**, how much do you agree with the following statements?
If the statement does not apply to you, if you are not sure, or if you are unfamiliar with the odours, please select the "not applicable" option.

| | I had phantom smells … | Not applicable | Disagree | Somewhat disagree | Neither agree nor disagree | Somewhat agree | Agree |
|---|---|---|---|---|---|---|---|
| 3 | … that are pleasant | ◻ | ◻ | ◻ | ◻ | ◻ | ◻ |
| 4 | … that are neutral | ◻ | ◻ | ◻ | ◻ | ◻ | ◻ |
| 5 | … that are unpleasant | ◻ | ◻ | ◻ | ◻ | ◻ | ◻ |
| 6 | … such as smoke (for example cigarette or campfire smoke) | ◻ | ◻ | ◻ | ◻ | ◻ | ◻ |
| 7 | … that are vague in their intensity | ◻ | ◻ | ◻ | ◻ | ◻ | ◻ |
| 8 | … that are like other odours in their intensity | ◻ | ◻ | ◻ | ◻ | ◻ | ◻ |
| 9 | … that are intense | ◻ | ◻ | ◻ | ◻ | ◻ | ◻ |
| 10 | … more often when I am disgusted (the disgust comes *before* the phantom smell) | ◻ | ◻ | ◻ | ◻ | ◻ | ◻ |
| 11 | … more often when I am indoors than when I am outdoors | ◻ | ◻ | ◻ | ◻ | ◻ | ◻ |
| 12 | … at least once an hour | ◻ | ◻ | ◻ | ◻ | ◻ | ◻ |
| 13 | … at least once a day | ◻ | ◻ | ◻ | ◻ | ◻ | ◻ |
| 14 | … at least once a week | ◻ | ◻ | ◻ | ◻ | ◻ | ◻ |

We are interested in the experience of odours that smell different than before. By *different* , we mean a change in pleasantness and/or quality, not a reduction or loss of smell. For example, coffee may smell unpleasant or unfamiliar, or "bathroom odours" smell less unpleasant than before. During the **past two weeks**, how much do you agree with the following statements?
If the statement does not apply to you, if you are not sure, or if you are unfamiliar with the odours, please select the "not applicable" option.

| | Some odours smelled different from what they used to … | Not applicable | Disagree | Somewhat disagree | Neither agree nor disagree | Somewhat agree | Agree |
|---|---|---|---|---|---|---|---|
| 15 | … as they have changed in character or quality | ☐ | ☐ | ☐ | ☐ | ☐ | ☐ |
| 16 | … as they are more pleasant than before | ☐ | ☐ | ☐ | ☐ | ☐ | ☐ |
| 17 | … as they are more unpleasant than before | ☐ | ☐ | ☐ | ☐ | ☐ | ☐ |
| 18 | … such as peanuts | ☐ | ☐ | ☐ | ☐ | ☐ | ☐ |
| 19 | … such as fish | ☐ | ☐ | ☐ | ☐ | ☐ | ☐ |
| 20 | … such as tomato | ☐ | ☐ | ☐ | ☐ | ☐ | ☐ |
| 21 | … such as chocolate | ☐ | ☐ | ☐ | ☐ | ☐ | ☐ |
| 22 | … such as meat | ☐ | ☐ | ☐ | ☐ | ☐ | ☐ |
| 23 | … such as lemon | ☐ | ☐ | ☐ | ☐ | ☐ | ☐ |
| 24 | … such as coffee | ☐ | ☐ | ☐ | ☐ | ☐ | ☐ |
| 25 | … such as eggs | ☐ | ☐ | ☐ | ☐ | ☐ | ☐ |
| 26 | … such as melon | ☐ | ☐ | ☐ | ☐ | ☐ | ☐ |
| 27 | … such as cucumber | ☐ | ☐ | ☐ | ☐ | ☐ | ☐ |
| 28 | … such as onion | ☐ | ☐ | ☐ | ☐ | ☐ | ☐ |
| 29 | … such as toothpaste | ☐ | ☐ | ☐ | ☐ | ☐ | ☐ |
| 30 | … at least once an hour | ☐ | ☐ | ☐ | ☐ | ☐ | ☐ |
| 31 | … at least once a day | ☐ | ☐ | ☐ | ☐ | ☐ | ☐ |
| 32 | … at least once a week | ☐ | ☐ | ☐ | ☐ | ☐ | ☐ |
