# Supplementary material for: Experts’ content validation of the parosmia, phantosmia, and anosmia test (PARPHAIT): A qualitative study
Source: PLoS One. 2025 Aug 5;20(8):e0329108. doi: 10.1371/journal.pone.0329108 (PMC12324124; doi:10.1371/journal.pone.0329108)
Supplement: S1 Table — (DOCX) [file pone.0329108.s005.docx]

**S1 Table. Codes, sub-codes, and illustrative quotes**

| **Code** | | | **Sub-code** | | **Illustrative quote** | |
| --- | --- | --- | --- | --- | --- | --- |
| **1** | Applicability | **1** | | Administration | | *[…] it's always nice to have a paper version, that's one thing, and then you need to have a digital version. So both of it would be nice (ID-12).* |
|  |  | **2** | | Patients’ own insight | | *[…] sometimes if you don't specifically ask the question to patients, they don't report the complaint spontaneously. So yes, I think it's interesting (ID-1).* |
|  |  | **3** | | Relevance of items | | *I never smell mowed grass because I live in a city. I don't pass flower shops. I don't have a cellar. So, yeah, it's just stuff like that where it's like, I don't know, maybe change it to stuff you know people are going to experience everyday, like, you can't smell your food or you can't smell your own toilet or something like that (ID-11).*  *Some patients just go “Well, I can smell this chemical smell”. They don't describe it as pleasant or unpleasant, it's just they're aware that there's an odour sensation that they can't find a source for. So I think having neutral is an appropriate question (ID-4).*  *[…] I remember several patients talking to me about the difference between the lemon they remember before their problem and the new lemon that they experience now […] They talk about a “new lemon”, so that's the thing, they're going to say “I know it's lemon but it doesn't smell like it used to, but I can recognise it enough to discern that it's lemon” (ID-4).*  *There is also the issue of hedonic versus qualitative changes in parosmia. My suspicion is that these can be independent. Could your questionnaire pick up on that? (e-mail).*  *“When I'm disgusted” is interesting. And I think it's very hard for people to respond accurately. Because I think they are disgusted because of the smell. And so whatever comes first (ID-13).* |
|  |  | **4** | | Time frame | | *I think parosmia is a kind of black swan event in people's lives. You know, It's like such shock that they remember clearly everything about it because, you know, they almost have post-traumatic stress disorder (ID-2).* |
|  |  | **5** | | Use in clinical setting | | *That can be helpful for an ENT. Because that would take time in conversation. And, you know, if the person is clearly parosmic, he just needs to confirm. And, you know, they can ask more tailored questions. So that I would see as helpful. It would save them time (ID-13).*  *[…] there really is nothing like you said for phantosmia. […] It's something we just come across clinically much, much less frequently. And so the amount that's devoted here would be great from a flow logic standpoint (ID-6).*  *Cause right now it's, as you're probably more aware than I am, very much a black box for how patients present this phantosmia. Typically, you have a burning chemically odour, or is it something that's pleasant in some cases and how often is it euosmia? We don't really know, and it's not captured. So, I think that would be a definite value to add from where we are (ID-6).* |
| **2** | Clarity | **6** | | Formulation of items | | *[…] people might interpret these questions in different ways from what you interpret them. If you say, “I don't perceive the musty odour in a damp cellar”, you know, people might interpret the word perceive differently, right? Some people might interpret that to mean, well, sometimes when I walk into a musty cellar, I actually feel the moisture, and that's part of my perception, right? And even though you're saying odour, the normal person on the street isn't necessarily discriminating amongst these different characteristics of their experience […] (ID-8).*  *I would put “when I'm disgusted” first […] So, the accent of disgust comes first and then I notice the smell. And then I would ask “When I perceive phantom smells, I find myself more disgusted”. You can try both. And if you have both, you can see a little bit of directionality when you compare the responses (ID-13).* |
|  |  | **7** | | Introductory text and symptom definitions | | *It might help to put people in a frame of mind for these first questions. “I'm going to ask you to picture yourself in different situations”. Because that helps people focus on the type of question that they're going to be having. And this is really kind of, okay, now I've got to think about if I was in a flower shop or if I'm in a different kind of environment. […] Sort of give them a place to put their expectations (ID-8).*  *[…] you want to put people in the condition to really understanding what you're trying to measure, because we're not used to making these distinctions. It's not that you can rely on people's self-knowledge about parosmia (ID-13).* |
|  |  | **8** | | Accessibility | | *There are some surveys where they use icons. You know, people who people find that much more user friendly. Yeah, rather than seeing the words on the page (ID-2).* |
|  |  | **9** | | Domain or component | | *You're capturing the hedonicity rather than the perception of the odour in that one [item 2], “I do not mind the bad smell”. It's not necessarily whether you can perceive it or not, it's just whether it bothers you (ID-10).* |
| **3** | Format | **10** | | Length | | *[…] the bad smell of public toilets and the dog pile smell are clocking the same thing. So you could remove one of those (ID-2).* |
|  |  | **11** | | Developing the scale | | *I think you just need to have a clinical… Or you need to try it out clinically in real people. Then you get much more information. You can think on your desk about things only for that long. You need to try it out, that would be my advice (ID-12).* |
|  |  | **12** | | Response design | | *I assume if you don't have phantosmia, the patient should check the “not applicable” column. And for me, it's not the same as neither agree nor disagree, because for me, that means if you answer this question, that by definition, you have phantosmia (ID-1).*  *Patients do all sorts of things. Draw little pictures, they do something, you know, the overdramatic ones, or put it right off the scale somewhere else. They're going to do all of that anyway. But if you're looking at, like you said, you want to make this as compact as possible. If you are considering moving away from just single answer form, then this is a way of doing that (ID-10).* |
|  |  | **13** | | Scoring design | | *Especially if you're doing a weekly thing, of course. A week is seven days. So, if you go from once a day to once a week, would that be a seven-unit step or should that be a one-unit step? … I mean, I suppose addition of time and severity might, you know, give you some use. Because as I said, if something's bad but infrequent, it's probably the same as something that's not bad but frequent (ID-10).* |
|  |  | **14** | | Structure | | *It depends on what you're hoping to achieve, if it’s a sort of global assessment of olfactory dysfunction, then combining them is appropriate. If you're wanting to get a sort of specific qualitative assessment, then obviously you need to separate them out, and the trouble is these things are not necessarily mutually exclusive, because, you know, some patients may have complete anosmia and phantosmia, some patients may have hyposmia and parosmia, some patients may describe things that sort of… You're not quite sure whether they're phantosmia or parosmia (ID-4).*  *It's a good idea in the sense that clinicians are really, really busy people, and it's complete chaos and… To give one piece of paper rather than three is always a good idea (ID-9).* |
| **4** | Aspects not covered | **15** | | Potential items | | *[...] it is true that a lot of post-viral anosmics will be able to perceive something, but they don't perceive it as unpleasant. Especially feces and urine, they don't, or body odour. They can say something there. They often describe it as biscuity or pleasant (ID-10).* |
|  |  | **16** | | Control questions | | *It's important though to probably have some anchors that wouldn't trigger parosmia. […] Everybody should universally be like, no, that's actually not a problem. Like, I don't know, water or something (ID-6).* |
|  |  | **17** | | Quality of life | | *Coffee doesn't smell like it used to, but it doesn't smell disgusting to them. That is not captured, or the difference between them and the people who are so parosmic they can't drink coffee is not captured in these questionnaires. And that's, I would argue, a very important thing. […] That's an impact on quality of life and something that you want to do something about. Somebody just goes “oh, you know, eggs don't quite smell like they used to”. That's completely different to “I vomit whenever I walk into a room that somebody's been preparing eggs” (ID-10).* |
|  |  | **18** | | Screening questions | | *So, I wonder if you consider including a general kind of a branching question. So, “Have you ever experienced phantom smells, or any vivid description of it?” Yes, no, don't know. And then you can remove all of the questions if people have never experienced that. So maybe you can keep the granularity for whom this is relevant, and you don't bother the others (ID-13).* |
