# Supplementary material for: Experts’ content validation of the parosmia, phantosmia, and anosmia test (PARPHAIT): A qualitative study
Source: PLoS One. 2025 Aug 5;20(8):e0329108. doi: 10.1371/journal.pone.0329108 (PMC12324124; doi:10.1371/journal.pone.0329108)
Supplement: S2 Table — (PDF) [file pone.0329108.s006.pdf]

S2 Table. Changes applied to PARPHAIT after patient content validation[a], exploratory factor analysis (EFA)[a], and expert content validation.

Codes	Sub-codes	Initial pool[a]	Patient content validation[a]	EFA[a]	Expert content validation
Applicability	Relevance			Removed 59 items	Added 3 items about change in character and valence, removed 4 items (retro/orthonasal and food triggers)
	Time frame	Added "past week"			Changed "past week" to "two past weeks"
Clarity	Formulations of items	Reformulated 17 items, added visual stimuli, temperature, emotional stimuli, and food triggers, removed 17 items.			Added sub-header to each sub-scale, compressed 6 quantitative items into 2 items covering similar constructs, reformulated all phantosmia sub-scale items and

Codes	Sub-codes	Initial pool[a]	Patient content validation[a]	EFA[a]	Expert content validation
					parosmia trigger sub-scale items. Frequency questions for the phantosmia and parosmia sub-scales were given the same formulations.
	Introductory text and symptom definitions	Reformulated introductory text for quantitative sub-scale. Added introduction, definition, and examples to phantosmia and parosmia sub-scales. Clarified the meaning of "not applicable".			Changed the introductory text for all sub-scales
	Accessibility		Added colous (grey, light blue,		Increased font size from 10 to 12

Codes	Sub-codes	Initial pool[a]	Patient content validation[a]	EFA[a]	Expert content validation
			and light yellow) to each sub-scale		
Format	Length				Reduced the number of items by combining two or more items into one
	Response design	Changed "Unfamiliar with the odour/Not relevant" to "Not applicable"			Moved "agree" to the right-hand side, "disagree" to the left.
	Structure			Was re-structured according to factors suggested after the EFA	Presents "I have phantom smells…" as a sub-heading instead of repeating it for every item. Suitable sub-headings were also added to the quantitative and parosmia sub-scales. The order

Codes	Sub-codes	Initial pool[a]	Patient content validation[a]	EFA[a]	Expert content validation
					of items was sorted according to aspects. In the phantosmia sub-scale the order is valence, intensity, emotional triggers, environmental triggers, and frequency. For the parosmia sub-scale the order is character/valence, food triggers, and frequency.
Aspects not covered	Potential items				Added 2 triggers: (cucumber and toothpaste) and smoke as a specific symptom to the phantosmia sub-scale.

[a] = Initial pool, patient content validation, and EFA are processes that are part of a previous study [1]. Changes at these stages were thus done prior to the current study.

References

1. Espetvedt A, Brønnick KK, Wiig S, Myrnes-Hansen KV, Lungu DA. Capturing qualitative olfactory dysfunction with PARPHAIT: the parosmia, phantosmia, and anosmia test. Rhinology Online. 2024;7:39-65.
